# Structuring an event ontology for disease outbreak detection

**DOI:** 10.1186/1471-2105-9-S3-S8

**Published:** 2008-04-11

**Authors:** Ai Kawazoe, Hutchatai Chanlekha, Mika Shigematsu, Nigel Collier

**Affiliations:** 1 National Institute of Informatics, 2-1-2 Hitotsubashi, Chiyoda-ku, Tokyo, 101-8430, Japan; 2 National Institute of Infectious Diseases, 1-23-1 Toyama, Shinjuku-ku, Tokyo, 162-8640, Japan

## Abstract

**Background:**

This paper describes the design of an event ontology being developed for application in the machine understanding of infectious disease-related events reported in natural language text. This event ontology is designed to support timely detection of disease outbreaks and rapid judgment of their alerting status by 1) bridging a gap between layman's language used in disease outbreak reports and public health experts' deep knowledge, and 2) making multi-lingual information available.

**Construction and content:**

This event ontology integrates a model of experts' knowledge for disease surveillance, and at the same time sets of linguistic expressions which denote disease-related events, and formal definitions of events. In this ontology, rather general event classes, which are suitable for application to language-oriented tasks such as recognition of event expressions, are placed on the upper-level, and more specific events of the experts' interest are in the lower level. Each class is related to other classes which represent participants of events, and linked with multi-lingual synonym sets and axioms.

**Conclusions:**

We consider that the design of the event ontology and the methodology introduced in this paper are applicable to other domains which require integration of natural language information and machine support for experts to assess them. The first version of the ontology, with about 40 concepts, will be available in March 2008.

## Background

**Figure 1:**
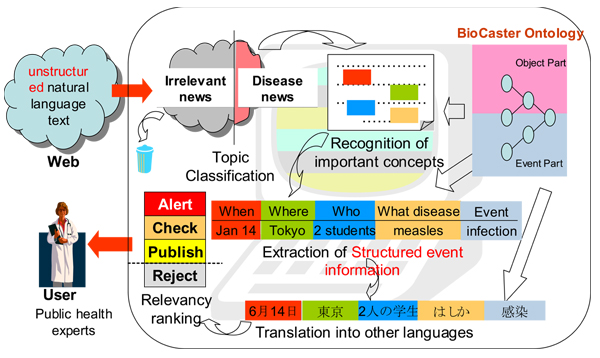
**An overview of the BioCaster disease surveillance system.** The system downloads news articles from online news feeds every few hours and filters out irrelevant topics. On the filtered articles, mentions to important concepts (disease cases, pathogens, time, location, etc) are recognized, and structured event information will then be retrieved. The information will be translated into other languages if necessary, and ranked according to its urgency and seriousness.

Timely detection of disease outbreak events and rapid judgment of their alerting status are the cornerstone of defence against the threat of infectious diseases. Today, it is estimated that about 65% of the initial reports on disease outbreaks come from news articles and informal information sources on the Web [1]. However, it is time-consuming work to find relevant reports from the vast amount of information, and thus efficient machine support is needed. In the BioCaster project [2], a text mining-based system is being developed to monitor disease outbreak reports, and to support public health experts in accessing, finding, and evaluating the information (Figure 1). As a component of the system, an ontology which includes event concepts will become the basis for machine understanding of disease outbreak information. Proper descriptions of event concepts and their linguistic realizations are necessary, since ‘event’ is a type of information which plays a central role in disease surveillance, and it is mentioned in a more complex way than object concepts.

This paper presents a rationale and design of the multi-lingual event ontology. This complements the extant BioCaster Ontology for objects [3], which is already released for object concepts and now in use within the BioCaster health alerting system [2]. The event ontology, which we refer to as ‘the BioCaster Event Ontology (BCEO)’, describes disease-related events such as outbreak of newly emerging infectious diseases, unusually severe cases of known infectious diseases and drug-resistant cases. It will be applied for language-oriented tasks, including identification, translation and integration of disease-related information in texts. This application ontology is designed to organize event concepts in a way to solve the following issues:

• **Bridging the gap between layman's languages and public health experts' knowledge**. Disease outbreak reports on the Web such as news articles are often written in non-technical language, while public health experts assess outbreak events with highly technical knowledge. It is necessary to provide them with information on the Web in a form which conforms to their own knowledge.

• **Making information available in multiple languages**. Usually the earliest information on diseases is reported in the local language. There is a need for access to foreign language information translated into the expert's first languages.

In this paper, we use the term ‘event’ as having the same meaning as perdurants, or occurrences in philosophy, or eventualities used in linguistics [4]. Although ‘event’ is often used to refer to telic occurrences, our use includes atelic ones too.

## Construction and content

BCEO can be regarded as a domain ontology for a particular purpose. It is not a domain ontology in the ordinary sense, since it does not have a detailed knowledge model. The application orientation impacts on the design in at least three ways: 1) the level of granularity, 2) scope (type of events described) and 3) multi-linguality.

### Important events and relations

We identified the following events which public health experts seek through the news articles available online. These are key events in disease news reports, and we desire to recognize the relevant linguistic expressions.

• Disease outbreaks (group infection)

• Infecting events in individuals

• Health status of patients (being sick, being hospitalized, being dead, etc)

• Control efforts to contain diseases (killing, treating, hospitalizing, etc)

We found that when the experts judge the seriousness of disease events in news reports, they look at the following aspects. Most of them are the properties of participants of events (i.e., infectious agents, etc).

• Kind of infected organism: animal infection, human infection

• Number of patients: more cases are considered more relevant.

• Location: outbreak in endemic area, non-endemic area, nosocomial outbreak

• Transmission route: animal-to-human, human-to-human

• Infectious capacity of pathogen

• Virulence (morbidity) of pathogen

• Potential for use in bio-terrorism

• Drug resistance of pathogen

Correlations of these aspects are taken into consideration to estimate the urgency and abnormality of events. For example, a single case of a virulent disease such as smallpox is more serious than a cluster case of a less virulent disease, and the first occurrence of an animal disease in humans is more important than its re-occurrence in animals. It is desirable that the surveillance system can automatically judge the relevance of events and alert the experts once a dangerous situation occurs. We consider that this can be partly achieved by appropriate modelling of knowledge in the event ontology combined with the rules that are customized to the interests of individual public health workers.

### Layman's language issue

Although public health experts look at disease-related events with technical knowledge as described above, reports on the Web are usually written in non-technical language, by non-experts. Not many technical terms appear in news articles compared to more professional publication types, and sometimes their meanings are generalized, vague or inaccurate. This makes it challenging to apply regular medical ontologies using traditional indexing strategies.

In many cases, disease-related events are mentioned with verbs (e.g. ‘infect’), verbal nouns (e.g. ‘infection’) and verb phrases. We also find many synonymous event expressions: e.g., infecting events can be expressed by many verbs and verb phrases such as ‘infect’, ‘transmit’, ‘contract’, ‘communicate (pathogen/disease)’, ‘catch (pathogen/disease)’, ‘get (pathogen/disease)’, verbal nouns such as ‘infection’, ‘transmission’, ‘contraction’. There are also cases such that an infecting event is not directly mentioned, but only implied, as in sentences like “A man died of bird flu”.

Participants of those events, such as disease cases, pathogens, source of infection, time and location are also mentioned in various ways. Sometimes they occur as arguments of verbs or verbal nouns which denote events, but in other cases they are expressed as non-argument modifiers (adjuncts). We also observe that sometimes an event is mentioned in one sentence and some of its participants are mentioned in another. Regarding the variety and complexity of event expressions, we consider the description of relations between relations (entailment, implication) and those between expressions (synonymy, etc) in an ontology to be beneficial in avoiding loss of data.

### Cross-language issue

It is important for any disease surveillance system to process multi-lingual information in order to detect earliest outbreak reports. In fact, some extant disease surveillance systems already provide a multi-lingual capability. Good examples are GPHIN [165], which supports the United Nation's official languages, and MedISys [6], which covers all the EU languages. However, language coverage is limited particularly in Asia-Pacific languages. The initial target languages of the BioCaster system are mainly Asian languages, which include Chinese, English, Japanese, Korean, Thai, and Vietnamese. When considering event mentions in multiple languages, formal definitions of events become critical. Since events are often mentioned by verbs, which usually have more than one meaning, simple translation may cause ambiguities and disagreements in definition of concepts. Metalinguistic definitions in ontology language and logic will help to avoid problems.

### Basic design

**Figure 2:**
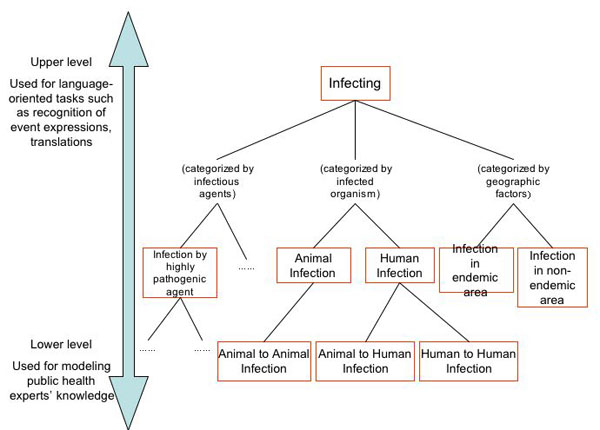
Basic design of the BioCaster Event Ontology

The needs and the challenges described above motivated us to develop an ontology which has 1) a model of public health experts' knowledge, 2) a description of relations between expressions which denote disease-related events in multiple languages, 3) formal definitions of event concepts. The basic design of BCEO is shown in Figure 2. It aims to cover disease-related event mentions in ordinary, layman's language by locating classes of rather general events in the upper-level. At the same time, experts' knowledge for disease surveillance is modelled in the lower level of the ontology, with additional restrictions on the participants of the events. The upper level can be applied for language-oriented tasks while the lower level can serve as a basis for assessing disease outbreak events.

### Taxonomy

**Figure 3:**
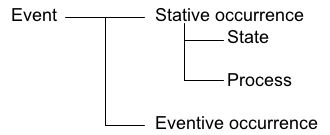
Top-level classification of event classes

**Figure 4:**
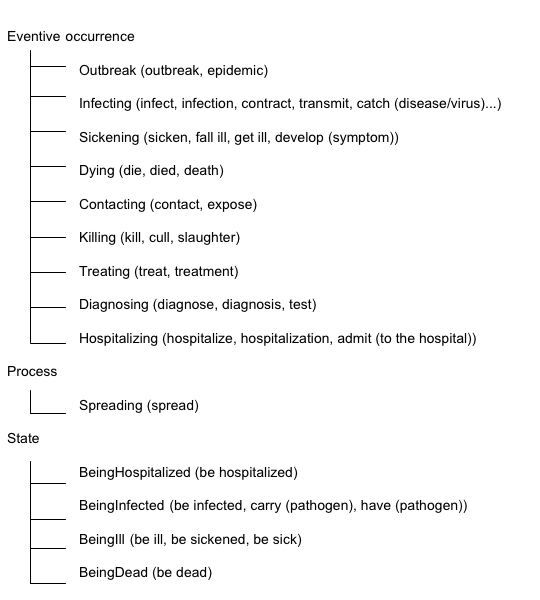
Event classes immediately under Eventive occurrences, Process and State.

We adopted some parts of DOLCE [7] as a basis for our top-level classification (we will describe the reason in the Discussion section). DOLCE's classification of perdurants is based on several formal properties (definitions are found in [8], p.21). An event is a stative occurrence when it is cumulative (i.e., the mereological sum of two of its instances is still the same type of event [9], [10], such as sitting and running), otherwise it is an eventive occurrence. Furthermore, a stative occurrence is a state if it is homeomerous (i.e., every part of that event is still the same type of event [11]; for example, sitting is homeomerous), otherwise it is a process (running for example is not homeomerous since some parts of running are different from other parts). We adopt the distinction between a Stative occurrence and an Eventive occurrence and the distinction between a State and a Process. The top level of BCEO is shown in Figure 3. We use a set of tests for classifying events into State, Process and Eventive occurrences, including the ones proposed by [12] and [13]. This has the advantage that we can apply the test to new verbs and events when we extend the ontology. The resulting upper-level classification trees are shown in Figure 4. Some corresponding expressions are shown between parentheses.

Further classification of events is done by breaking down the upper event classes by adding more restrictions on their participants (e.g., the class Infecting has a subclass Human Infection, which imposes an additional restriction on the *theme* participant). This process will produce a plurality of perspectives as shown in Figure 2.

### Description of event classes

**Figure 5:**
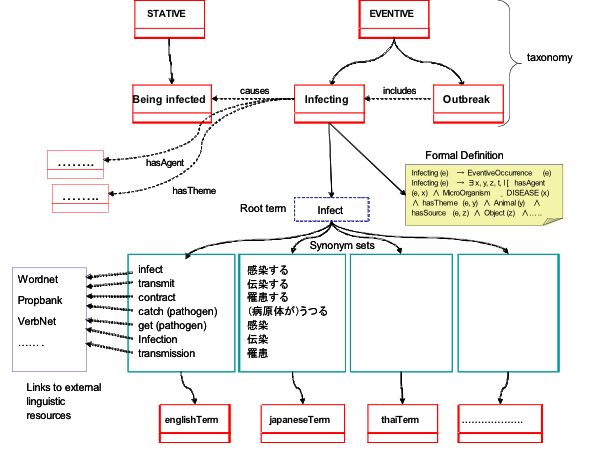
A closer look at an event class

A closer look at the design of BCEO is shown in Figure 5. There we can see relationships which involve event classes (causes, includes, hasAgent, hasTheme), links from an event class to multilingual synonym sets via a root term, a link to the formal definitions, and links between each synonym and external linguistic resources.

### Properties

BCEO aims to cover important relations 1) between an event and its participants and 2) between an event and another event (*includes* and *causes*). The former includes relations between an event and its agent, theme, source, location and time, etc, which are realized as OWL properties such as *hasAgent, hasTheme, hasSource, starting/endingLocation*, and *starting/endingTime*. It should be noted that “participants of events” here should be distinguished from arguments of verbs. The *includes* property is used to describe the relation between a macro-event and its sub-events, for example, the inclusion of a single infecting event within an outbreak (group infection) event. We follow [14] in assuming an outbreak event is a kind of macro-event (defined in [15]), which includes infecting events or smaller outbreak events as its sub-events. The importance of the inclusion relation among outbreak events in Information Extraction is discussed in [16]. *Causes* describes a causative relation between events, such as a causation of an infected state by an event of infection, or the cause of death by infection. *Includes* and *causes* are important in establishing entailment relations between linguistic expressions, to enable inferences (e.g. from “There was an outbreak of avian influenza in humans” to “At least one person was infected with avian influenza”, or from “A person died of avian influenza” to “A person was infected with avian influenza”). This will serve to complement incomplete information found in texts.

### Axioms

It should be noted that the aim of the ontology is a conceptualization of disease-related events, and NOT a classification of verbs. Formal description of event classes, including those which cannot be expressed with OWL properties, are given as axioms in event logic (e.g. anteroposterior relationships between events in a *causes* relation, restriction on time and location of events accompanied by *includes* relation). Following is an axiom which represent a necessary condition for the Infecting class.

• Infecting (e) ∧ hasTheme (e,x) ∧ endingTime (e,t) ∧ endingLocation (e,l) → ∃e′ [causes (e,e') ∧BeingInfected (e') ∧ hasTheme (e′,x) ∧ startingTime (e′,t) ∧ startingLocation (e′,l)] (An Infecting event induces a BeingInfected state)

### Synonym sets

Each event class is linked to sets of synonymous terms via a *root term*, which serves as an interlingual pivot. The synonym sets are different from synsets in WordNet [17][18] in that 1) any expressions which can refer to the same class of events can be included in the same set regardless of their syntactic categories, 2) verbs or verbal nouns which have different argument structures can belong to the same set (however, information of subcategorization is provided for each entry of the synonym set, via linkages with external linguistic resources such as PropBank [19] and WordNet). We use the following test to determine if two expressions can be members of the same set.

1. Make two sentences from the verbs (for verbal nouns, sentences started as “There is/was an VN.…”), adding the same participants, time, and location to both.

2. See if there is a situation where you can affirm one while negating the other, and vice versa. If you cannot find any, the two expressions are synonymous.

Let us see an example. Suppose we would like to see if the two verbs, ‘contract’, and ‘infect’ are synonymous. Following part 1 of the test, we construct sentences “Mr. A contracted influenza virus from Mr. B on Jan 15 in Oedo-city”(S1) and “Mr. B infected Mr. A with influenza virus on Jan 15 in Oedo-city”(S2), providing each of the verbs with the same participants, time and location. Then we see if we can affirm S1 while negating S2 as in “Mr. A contracted influenza virus from Mr. B on Jan 15 in Oedo-city, but Mr. B DID NOT infect Mr. A with influenza virus on Jan 15 in Oedo-city”(S3) and vice versa as in “Mr. A DID NOT contract influenza virus from Mr. B on Jan 15 in Oedo-city, but Mr. B infected Mr. A with influenza virus on Jan 15 in Oedo-city”(S4). We can see that neither S3 nor S4 can be true in any situation, thus the two verbs are synonymous. Although this example is monolingual, this test can be applied to multi-lingual texts on condition that there is a person who can make the linguistic judgment in both languages.

## Utility and discussion

### Application of BCEO

BCEO described so far will be applied for 1) annotating/grounding text mentions of events (i.e., establishing linkages between events and their linguistic realizations), 2) translation of terms which denote events, 3) modelling of public health experts' knowledge used to judge the alerting relevance of disease-related events. (1) is important in making a basis for automatic recognition of varieties of event expressions in disease outbreak reports, in addition to the traditional named entities. (2) is necessary to provide disease outbreak information in users' native languages, and grasp relationship (identity, overlap, causation) between reports written in different languages. In BCEO, multi-lingual synonym sets will be used for these purposes. While (1) and (2) are rather language-oriented aspects, (3) is a knowledge-oriented aspect. The lower-level event classes, along with their OWL properties and formal definitions in axioms can be utilized for describing the knowledge.

### Relationship with existing ontologies

We now compare how disease-related event concepts and event expressions have been covered in BCEO to some of the existing ontologies.

PHSkb [20] is a coding system that has been developed to support the exchange of electronic data about observations of notifiable diseases between public health experts in the United States. It includes a list of events which experts find reportable, such as outbreaks. Although this can be seen as a model of public health experts' knowledge, terms listed there (for example, “nosocomial outbreak” or “common source outbreak”) do not often appear in news texts. Also the classification incorporates many points of differentiation, including experts' assessment of events (e.g. “unusual”), while the validity of having such epistemic viewpoint in the ontology (as a model of reality) is still controversial. SNOMED CT [21] is a medical terminology collection based on description logic and has been extended to several languages including German and Spanish. Each concept is linked to a set of synonyms which includes nouns or noun phrases only, verbs and verb phrases are not covered. It has a rich list of event concepts related to disease surveillance, such as disease control measures and transmission modes, but group infection events are not modelled. ICD10 [22] is a detailed and widely used coding system for diseases published by the WHO. The classification is far more fine-grained than the level that is practical for terms that will appear in news sources, and it does not cover disease outbreak events which we aim to describe, i.e., a specific occurrence of a disease bound to time and place. Most of the biomedical ontologies and terminology systems including GALEN Core [23], and the UMLS [24], include event concepts with more technical descriptions for the use by experts in biomedicine, with a scope far broader and deeper than the task of disease detection and monitoring in Web texts. BCEO can be considered as having a role to play in filling the gap between terms which appears in the biomedical ontologies and those found in the news reports. We are also aware of the importance of links to existing resources. In the BioCaster Ontology for objects most concepts registered have links to ICD-10, MeSH and SNOMED CT, etc.

Linguistic expressions which refer to disease related events in outbreak reports are mostly covered by lexical resources such as WordNet [17][18] and EuroWordNet [25]. We take inspiration from EuroWordNet in several areas such as the use of a mediating node which we call a *root term*, in order to bridge sets of synonyms in different languages. These lexical resources are not intended to be domain specific and covers rather general and common event expressions. We referred to them when we expand our synonym sets which is related to more specific event concepts. PropBank [19] has a list of verbs with their arguments, and do not cover verbal nouns and common nouns. We link verbs in the BCEO synonym sets to corresponding PropBank entries to gain information about argument structures.

Top-level classification of events is provided by upper ontologies such as SUMO [26], BFO [27] and DOLCE [7][8]. Each of them classifies events from different perspectives, and it does not seem easy to reduce them into one perspective. As we discussed, we adopted DOLCE since it has established tests for classification, top-level classes disjoint to each other and has high affinity with classification of verbs.

As we have discussed, in order for an event ontology to be a basis for machine understanding of natural language information on disease outbreaks, it needs to include 1) a model of important disease-related events, 2) event expressions found in outbreak reports on the Web, 3) formal definitions of event concepts. Although each of the existing ontologies seems to meet a part of the requirements in the disease monitoring task, it is hard to find one which satisfies all of them. This calls for re-organizing knowledge and terminology in a way to integrate all three required aspects.

## Conclusion

We have introduced the design and construction of BCEO, which is being developed for application to multi-lingual text mining for disease surveillance. The event ontology integrates a model of experts' knowledge for disease surveillance, a structured vocabulary of linguistic expressions, and descriptions of event classes in meta-linguistic, logical representation. These features are essential to bridge between natural language texts and experts' deep knowledge. We consider that the design of the event ontology and the methodology introduced in this paper are applicable to other domains, and will be useful especially in those which treats emergency information such as chemical and nuclear incidents. Now we are developing the first version of the ontology with about 40 event concepts, linked to synonym sets in English and Japanese. The first version will be published online in March 2008.

## Competing interests

The authors declare that they have no competing interests.

## Author's contributions

This work was directed by NC. AK designed the ontology by extending the basic framework made by NC. HC analyzed 1000 online news articles and examined linguistic expressions which denote disease-related events in English. Japanese expressions are complemented by AK. MS identified important events and public health experts' knowledge which should be covered by the ontology. All authors contributed during the whole length of the ontology construction and writing of the paper. All authors read and approved the final manuscript.
